# Risk factors for schistosomiasis in an urban area in northern Côte d’Ivoire

**DOI:** 10.1186/s40249-018-0431-6

**Published:** 2018-05-18

**Authors:** Richard K. M’Bra, Brama Kone, Yapi G. Yapi, Kigbafori D. Silué, Ibrahima Sy, Danielle Vienneau, Nagnin Soro, Guéladio Cissé, Jürg Utzinger

**Affiliations:** 1 Unité de Formation et de Recherche des Sciences de la Terre et des Ressources Minières, Université Félix Houphouët-Boigny, 01 BP V 34, Abidjan 01, Côte d’Ivoire; 2 Centre Suisse de Recherches Scientifiques en Côte d’Ivoire, 01 BP 1303, Abidjan 01, Côte d’Ivoire; 3 Swiss Tropical and Public Health Institute, P.O. Box, CH-4002, Basel, Switzerland; 4 University of Basel, P.O. Box, CH-4003, Basel, Switzerland; 5 Institut de Gestion Agropastorale, Université Péléforo Gon Coulibaly, BP 1328, Korhogo, Côte d’Ivoire; 6 Centre d’Entomologie Médicale et Vétérinaire, Université Alassane Ouattara, 27 BP 529, Abidjan 27, Côte d’Ivoire; 7 Unité de Formation et de Recherche Biosciences, Université Félix Houphouët-Boigny, 22 BP 522, Abidjan 22, Côte d’Ivoire; 8 Centre de Suivi Ecologique, BP 15 532, Dakar, Senegal

**Keywords:** Côte d’Ivoire, Schistosomiasis, School-aged children, Urban agriculture, Vulnerability index, Water, Sanitation and Hygiene (WASH)

## Abstract

**Background:**

Schistosomiasis is a water-based disease transmitted by trematodes belonging to the genus *Schistosoma*. The aim of this study was to assess the relationship between the prevalence of schistosomiasis and access to water, sanitation and hygiene (WASH) and environmental and socioeconomic factors in the city of Korhogo, northern Côte d'Ivoire.

**Methods:**

A cross-sectional study including 728 randomly selected households was conducted in Korhogo in March 2015. The heads of the households were interviewed about access to WASH and environmental and socioeconomic factors. All children abed between 5 and 15 years living in the households were selected to provide stool and urine samples for parasitological diagnosis of *Schistosoma mansoni* and *Schistosoma haematobium* infection. The relationship between infection with *S. mansoni* and potential risk factors was analysed by a mixed logistic regression model with ‘household’ as a random factor. Likelihood ratio tests were used to identify factors that were significantly associated with a *Schistosoma* spp. infection.

**Results:**

The overall prevalence of schistosomiasis among school-aged children in Korhogo was 1.9% (45/2341) composed of 0.3% (3/1248) *S. haematobium* and 3.5% (42/1202) *S. mansoni*. Due to the low prevalence of *S. haematobium* infection, risk factor analysis was limited to *S. mansoni*. Boys were 7.8 times more likely to be infected with *S. mansoni* than girls. Children between 10 and 15 years of age were 3.8 times more likely to be infected than their younger counterparts aged 5-10 years. Moreover, living in a house further away from a water access point (odds ratio [*OR*] = 0.29, 95% confidence interval [*CI*]: 0.13–0.70) and abstaining from swimming in open freshwater bodies (*OR* = 0.16, 95% *CI*: 0.04–0.56) were significantly associated with decreased odds of *S. mansoni* infection. The socioeconomic status did not appear to influence the prevalence of *S. mansoni*.

**Conclusions:**

A strategy to reduce the incidence of schistosomiasis should focus on health education to change the behaviour of populations at risk and encourage communities to improve sanitation and infrastructure in order to reduce contact with surface water.

**Electronic supplementary material:**

The online version of this article (10.1186/s40249-018-0431-6) contains supplementary material, which is available to authorized users.

## Multilingual abstract

Please see Additional file 1 for translations of the abstract into the six official working languages of the United Nations.

## Background

Schistosomiasis is a tropical disease of humans and animals caused by a trematode worm which infects the host during water contact [1]. The vast majority (approximately 90%) of cases are reported from sub-Saharan Africa [2] where the two predominant disease sub-types are intestinal and urogential schistosomiasis caused by *Schistosoma mansoni* and *S. haematobium*, respectively [3, 4].

In some areas of West Africa and the Sahel that are marked by long periods of drought, several important irrigation systems were constructed during the International Decade of Drinking Water and Sanitation (1980–1990) in order to achieve food self-sufficiency and sustained water supply [5]. However, irrigation systems, such as small multipurpose dams, schemes for irrigated rice farming and shallows can lead to environmental disruptions that are difficult to foresee. The construction of a dam on the Bandaman River in Korhogo, northern Côte d’Ivoire [5], for instance, led to ecological changes that were linked to an increased risk in schistosomiasis [6–8].

Schistosomiasis in Côte d’Ivoire has a prevalence that ranges from less than 1% to over 90% depending on the social-ecological setting [5]. In 1997, the north of the country experienced a mean prevalence of almost 30% [5]. Transmission of schistosomiasis is also characterized by spatio-temporal variations [9]. People become infected during activities in open freshwater bodies such as agriculture, fishing and swimming [2, 10]. Some studies suggest that there is a lack of water, sanitation and hygiene (WASH) in areas where schistosomiasis is endemic [2, 10, 11] and there is a growing body of evidence suggesting that open defecation increases the risk of infection [12]. However, Erko et al. found that using soap containing endod can reduce the prevalence of schistosome infection [13]. Because WASH can depend on sociodemographic and socioeconomic factors, it is likely that WASH exerts an indirect effect on schistosomiasis transmission [14]. Based on their systematic review and meta-analysis, Grimes et al. recommended that new research on the link between WASH, human exposure and *Schistosoma* spp. infection rates is needed in order to provide design and implemant setting-specific interventions [15].

Korhogo is the largest and most populated city in northern Côte d’Ivoire. Deficiencies in water supply and sanitation are of major concern in the city despite efforts of the local authorities to improve this situation [16, 17]. Indeed, a significant proportion (63%) of the population continues to obtain drinking water from unprotected wells [16]. In general, the problem of access to water and sanitation is more critical in secondary cities of low and middle-income countries because of the lower basic infrastructure compared with the situation in the capitals. In addition, Korhogo has a long dry season with occasional heavy rains and flood events that could lead to serious health hazards because of an inadequate and poorly maintained sanitation infrastructure [18]. Many activities (e.g. agriculture, fishing, watering animals, washing clothes and swimming) are conducted around the man-made dam and in other open freshwater bodies in the city, which may enhance exposure to intermediate host snail of schistosomiasis.

There are a few studies in West Africa which have investigated the impact of access to water on neglected tropical diseases (NTDs) transmission [19], but none has specifically looked at the relationship between WASH and *S. mansoni* infection taking into account socioeconomic factors in the north of Côte d’Ivoire. Thus, the aim of this study was to assess the relationship between the transmission of *S. mansoni* and access to WASH in the city of Korhogo, taking into account environmental and socioeconomic factors to help to develop sustainable strategies to control schistosomiasis and other vector-borne diseases.

## Methods

### Study area

The study was carried out in Korhogo (05°38′19” W longitude and 09°27′41” N latitude), a secondary city in northern Côte d’Ivoire with an estimated 285 000 inhabitants [20]. The city lies within the Sudan type climate zone where seasons are controlled by the movement of the Intertropical Convergence Zone (ITCZ) [21]. Korhogo is characterised by two main seasons: a dry season from November to April and a rainy season from May to October, marked by two rainfall maxima in June and September, respectively. The average annual precipitation varies between 1000 mm and 1300 mm and the average annual temperature is 27 °C [22]. The vegetation is characteristic of the west Sudan Savannah type. Korhogo is largely drained by the Bandama River and its tributaries. A drinking water supply dam was built in Korhogo in 1981 with a capacity of 10^7^ m^3^. The water of the dam is treated to make it suitable for drinking and is distributed through a network of channels to households in the city by the water distribution company in Côte d’Ivoire (Société de Distribution d’Eau en Côte d’Ivoire [SODECI]).

### Study design and sample size calculation

A cross-sectional study was conducted in Korhogo in March 2015 during the dry season to determine the environmental and socioeconomic risk factors of schistosomiasis at the level of households and individual household members. A total of 728 households, distributed across the 29 neighbourhoods of the city, were randomly selected for a household survey. In addition, a parasitological survey was conducted, involving all school-aged children (5–15 years old) in the selected households, to determine the current status pertaining to *Schistosoma* spp. infection. Additionally, a geographical survey was conducted to identify and map the water access points and shallows in all neighbourhoods as well as in the peripheral zone of the city. Typical practices of the population at these water bodies were observed and registered.

The sample size was calculated by the formula [23, 24]:

$$ n=\frac{1.96^2\times \mathrm{P}\left(1-\mathrm{P}\right)\times \mathrm{C}}{i^2} $$

Assuming a prevalence rate of schistosomiasis of 0.35 (i.e. 35%) in the city [5, 25] and a design factor C for clustering of 2, with a statistical error < 0.05 (i.e. 5%) with 95% probability, a minimum number of 700 households was determined. To achieve a better distribution between neighbourhoods, based on their size, 726 households were randomly selected across the city.

### Data collection

The household survey was conducted by trained interviewers, and was administered to the household head or spouse. The survey included questions about access to WASH, socio-demographic characteristics of the household and about the socioeconomic status of the interviewee. Access to water was assessed to determine the main source of drinking water commonly used in the household and the sources of water for washing clothes and dishes and for gardening (among those who were gardeners). Access to sanitation and hygiene was assessed by the presence or absence of a latrine at household level and by the area where people discharge wastewater and solid waste. If there was a latrine, the interviewer documented, with the agreement of the interviewed person, whether it was modern or traditional (i.e. with or without flush) and the location of the latrine (i.e. inside or outside the house). If there was no latrine, the interviewee was asked to indicate the area where the members of the household defecate. We also collected information on the presence or absence of water bodies close to the house and the activities related to these waters, such as agricultural practices, fishing, swimming, laundry and crossing. The practices at water points were observed and the geographical coordinates of these points were registered using Global Positioning System (GPS) by the geographical survey team.

For sociodemographic status, we collected information concerning the household size, the education level and the main activity of the household head or spouse. We assessed the socioeconomic status by documenting any ownership of a car, motorcycle, television set, availability of electricity and house construction materials (i.e. mud/brick house, grass/iron or cemented roofs). The household head or spouse was interviewed in French or in one of the local languages (Sénoufo and Malinké). Households geographic coordinate were recorded using a hand-held GPS device.

A parasitological survey was performed for school-aged children of the same households to determine the prevalence and intensity of *S. mansoni *and *S. haematobium*. Prior to collecting samples, we obtained the parent’s written informed consent for all children. Moreover, children aged between 12 and 15 years assented. Children's age and sex were recorded, along with the educational attainment of the head of household. On the day of the household survey, information was given regarding stool and urine sample collection. Each participant received two labeled specimen containers of 125 ml each and was asked to provide one fresh morning stool sample (taken before 8 a.m.) and one urine sample (taken between 10 a.m. and 2 p.m.) the next day. On collection day, the parasitological survey team visited the household around 10 a.m. to collect samples for laboratory analysis. Urine samples were vigorously shaken and 10ml filtered for determining *S. haematobium* eggs under a microscope, while faeces were subjected to the Kato-Katz technique [26]. Two Kato-Katz thick smears were prepared from each stool sample and examined by two different technicians to detect and quantify *S. mansoni* eggs under a microscope.

### Data analysis

#### Statistical analysis

Data were entered using EpiInfo version 3.5.3 (Centers for Disease Control and Prevention; Atlanta, GA, USA)) and analysed using Stata version 14.1 (Stata Corporation; College Station, TX, USA). Ten percent of questionnaires were re-entered by another person for quality control.

Main outcomes of the study were the prevalence of *S. mansoni *and *S. haematobium*. Children were defined as being positive for *S. haematobium* and/or *S. mansoni* infection if at least one egg was detected in the 10ml filtrate of urine or any of the two Kato-Katz thick smears. The explanatory variables or potential risk factors assessed were access to safe water, type of latrine, mode of drainage of waste, sociodemographic factors, socioeconomic status, environmental factors (e.g. vicinity to water bodies), behaviours or practices of household members and their knowledge about the disease and risk factors. We used Fisher’s exact test to compare schistosome infection prevalence between groups. The relationship between cases of schistosomiasis and the aforementioned variables was analysed by a mixed logistic regression model with ‘household’ as random factor both with and without adjustment for socioeconomic characteristics and education level of the household head.

The likelihood ratio test was used to identify those factors that were significantly associated with schistosomiasis. Odds ratios (OR) with 95% confidence interval (*CI*) were used to measure the strength of associations. Statistical significance was defined at the level of 0.05.

Principal component analysis (PCA) was used to determine the vulnerability index of each neighbourhood based on environmental variables.

Scores were first defined at the household level and then averaged within a neighbourhood. Finally, neighbourhood means were divided into three categories using the k-means procedure.

#### Cartographic analysis

ArcGIS version 10.2 (ESRI; Redlands, CA, USA) was used to evaluate the spatial distribution of schistosomiasis cases and environmental risk factors. Type of latrine, source of drinking water and surface water bodies were mapped, and the spatial correlation between the distribution of schistosomiasis and explanatory variables were evaluated at neighbourhood scale (neighbourhood as geographic unit).

## Results

### Access to water, sanitation and hygiene

**Fig. 1:**
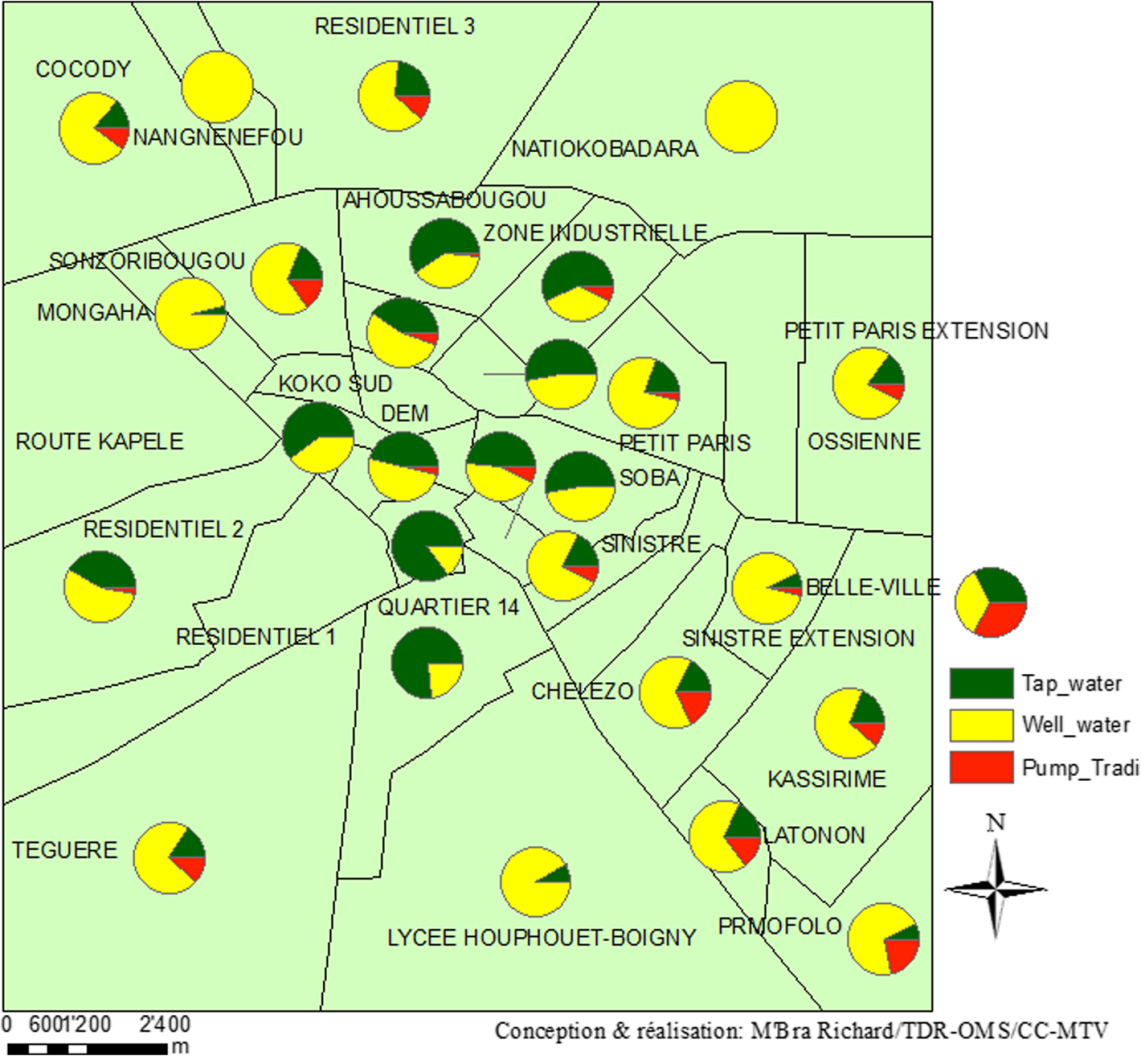
Geographical distribution and type of drinking water sources in the households of Korhogo, stratified by neighbourhood (March 2015)

The main surface water points and the geographical distribution of drinking water supply sources in the households are presented in Fig. 1. For drinking water, the majority of households reported to rely on well water (63.4%), while fewer used tap water (34.4%) and traditional pumps (5.6%). None of the surveyed households reported drinking directly from surface water.

**Fig. 2:**
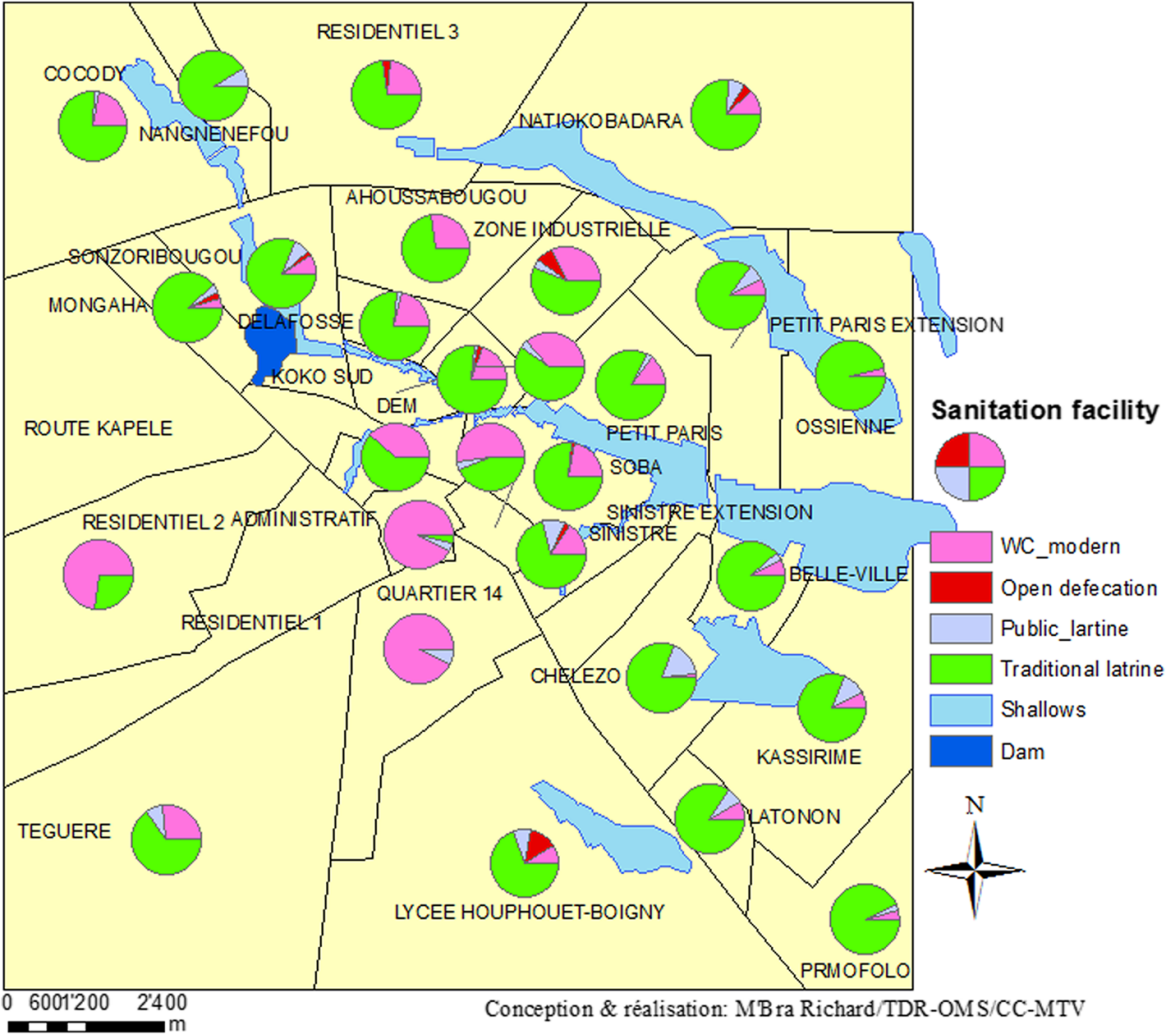
Geographical distribution and type of latrines used in the households of Korhogo, stratified by neighbourhood (March 2015)

Overall, 69.0% of the population reported to use traditional latrines vs. 24.7% using modern latrines. Substantially fewer (4.9%) relied on public latrines or practicing open defecation (1.4%) (Fig. 2). However, this distribution varied across neighbourhoods assessed.

**Fig. 3:**
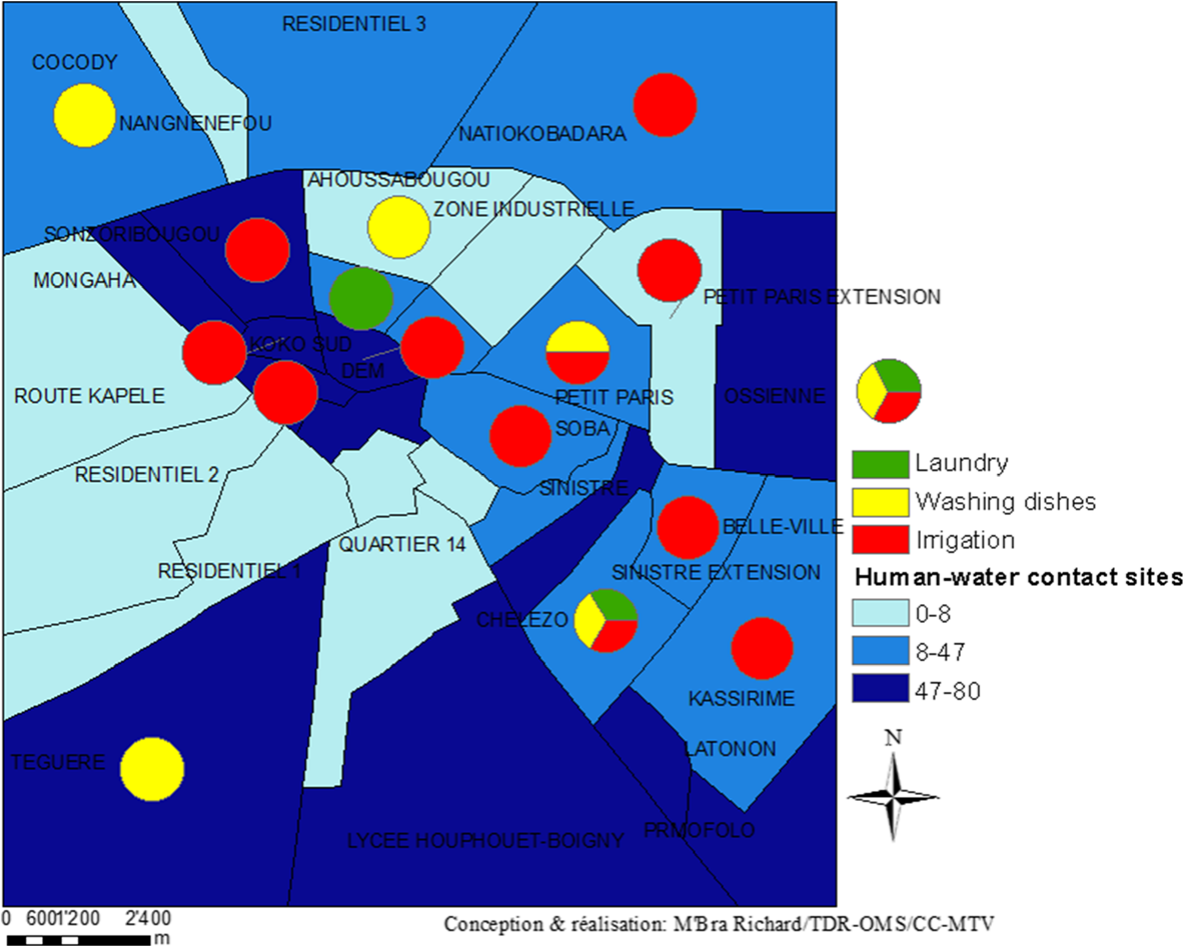
Number of human-water contact sites and main activities at these sites in Korhogo, stratified by neighbourhood (March 2015)

The activities carried out at the open freshwater bodies that were reported to be in close proximity to the households are presented in Fig. 3. Most of the households indicating close proximity to water bodies were from the neighbourhoods *Koko, Banaforo, Ossienne* and *Lycée Houphouet-Boigny.* The principal activities reported were irrigation, washing dishes and laundry.

Households with good access to tap water and sanitation were located in the neighbourhoods *Administratif, Résidentiel 1&2, Quartier 14* and *Air France.* Moreover, populations living in these neighbourhoods were located far from surface water points and generally did not participate in water-related activities (Fig. 3).

### Environmental vulnerability index of the neighbourhoods

**Fig. 4:**
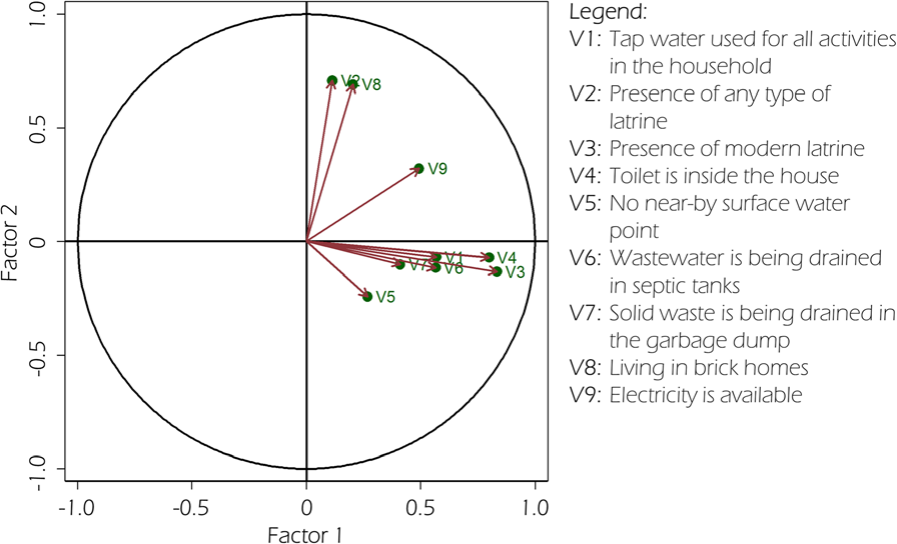
Principal component analysis of variables related to environmental vulnerability in the city of Korhogo (March 2015) in the F1-F2 plane. The x- and y-coordinates of a variable are given by its correlations with the first (F1) and second factor (F2), respectively

The level of environmental vulnerability of neighbourhoods of Korhogo was calculated using PCA based on environmental and socioeconomic variables selected. The relationships between the variables and factors are indicated by a correlation circle (Fig. 4).

**Fig. 5:**
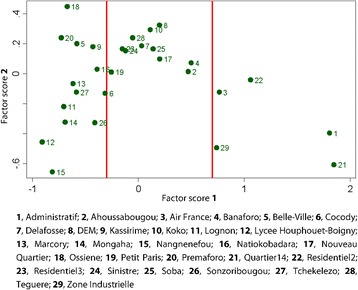
Mean values of the first (x-coordinate) and second (y-coordinate) principal component score by neighbourhood. The mean value of the first score expresses environmental vulnerability of the respective neighbourhood (low values indicate higher vulnerability). Red lines indicate boundaries between consecutive categories of environmental vulnerability (defined by the k-means procedure)

The first factor (F1) was defined by the variables ‘*tap water used for all activities*’ (*r* = 0.6), *‘presence of modern latrine’* (*r* = 0.8), *‘toilet is inside the house’* (*r* = 0.8), *‘electricity is available’* (*r* = 0.5) and *‘wastewater is being drained in septic tanks’* (*r* = 0.6). F1 represents the presence of an adequate health infrastructure and the implementation of good environmental practices at home. Factor 2 (F2) was defined by the variables *‘living in brick homes’* (*r* = 0.7) and *‘presence of any type of latrine’* (*r* = 0.7). According to the calculated vulnerability index, the assessed neighbourhoods of the city were allocated into three classes or three levels of vulnerability, as follows (Fig. 5): (i) class 1 represents the most vulnerable neighbourhoods, with z-score between − 1 and − 0.7 (i.e. with negative values for factors). They were identified as *Nangnenefou, Lycée Houphouet-Boigny, Lognon, Ossiéné, Sonzoribouou, Marcory, Premaforo, Belle-Ville, Cocody, Natiokobadara, Tchekelezo, Kassirimé* and *Mongaha*; (ii) class 2 represents neighbourhoods with an intermediate vulnerability level, with z-score between − 0.7 and 0.7 (most neighbourhoods had a positive score for F1 and a positive score for F2). These neighbourhoods were *DEM, Banaforo, Nouveau Quartier, Sinistré, Petit Paris, Soba, Delafosse, Koko, Teguere* and *Ahoussabougou*; (iii) class 3 represents the least vulnerable neighbourhoods with a z-score between 0.7 and 1.8. The strong positive correlation with F1 indicates that households in these neighbourhoods have a solid sanitary and environmental infrastructure and that the residents adopt good environmental practices. These neighbourhoods also exhibit a positive correlation with F2, reflecting that their houses were mostly brick homes and the absence of near-by surface water points. These were found to be Residentiel 2, Air France, Zone industrielle, Administratif and Quartier 14.

### Spatial distribution of schistosomiasis cases in Korhogo

**Fig. 6:**
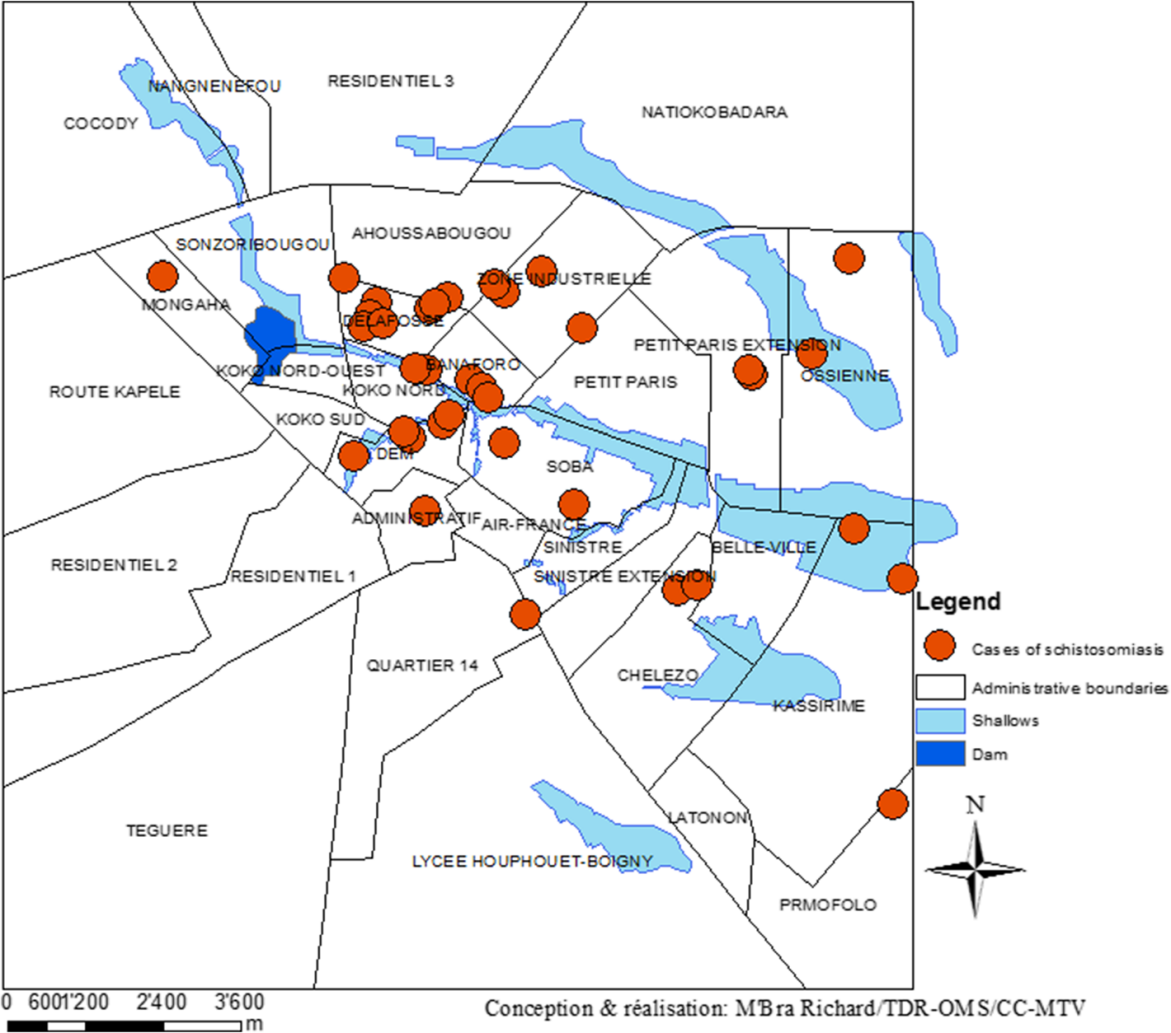
Spatial distribution of households with schistosomiasis cases in Korhogo (March 2015)

The overall prevalence of schistosomiasis among school-aged children in our study in Korhogo was 1.9% (45/2381), with 0.3% (3/1248) of urogenital schistosomiasis and 3.7% (42/1133) of intestinal schistosomiasis. Figure 6 shows the spatial distribution of households in the city of Korhogo where at least one case of schistosomiasis was found.

Most of the neighbourhoods with confirmed infections are of vulnerability class 1 and 2. *Delafosse, Ossiene, Belle-ville* and *Tchekelezo* have been listed among those with limited access to water and sanitation and located near water points.

### Knowledge and sociodemographic and environmental risk factors of *S. mansoni* in Korhogo

The knowledge of the population of Korhogo on schistosomiasis (transmission and symptoms) is described in Table 1. In our survey, we found that only 17.8% (129/724) of the interviewees knew about the disease. Washing in surface water and contact with waste water were mentioned as main causes of transmission by 70.6% and 53.3% of the interviewees, respectively. The presence of blood in urine and in stool was mentioned as the main symptoms of schistosomiasis by 68.5% and 54.8% of the respondents, respectively.

**Table 1:** Knowledge about schistosomiasis in the study population in Korhogo (March 2015)

Variables	Yes	Total
	*n* (%)	*n* (%)
Knowledge of the diseases		
blood in urine or stool	129 (17.8)	724 (100)
Is schistosomiasis transmitted by		
• Drinking unsafe water	209 (31.2)	671 (100)
• Eating contaminated food	13 (1.9)	
• Washing in surface water	474 (70.6)	
• By contamination	42 (6.3)	
• Walking without shoes in urine		
of infected person	42 (6.3)	
• Contact with waste water	358 (53.3)	
• Others	53 (7.9)	
Do you know the symptoms?	73 (71.6)	102 (100)
Is schistosomiasis manifested by?		
• Painful urination	16 (21.9)	73 (100)
• Urine scanty and frequent	7 (9.6)	
• Blood in stool	40 (54.8)	
• Intestinal disorders	1 (1.4)	
• Headache	3 (4.1)	
• Blood in urine	50 (68.5)	
• Others	13 (17.8)	

The sociodemographic characteristics most often associated with *S. mansoni* in Korhogo were sex (*P* < 0.001), age (*P* < 0.001) and education level (*P* = 0.011) (Table 2). The risk of infection for boys (7.0%) was 7.8 times higher than the risk of infection in girls (0.9%). Moreover, the children between 10 and 15 years of age (5.8%) were 3.8 times more likely to be infected than they younger by counterpart aged 5-10 years(1.5%).

**Table 2:** Sociodemographic characteristics and their association with schistosomiasis in Korhogo in (March 2015)

Characteristics	Infected *n* (%)	*P*-value
	*n* = 41	
Sex		
Males (*n* = 516)	36 (7.0)	< 0.001
Females (*n* = 581)	5 (0.9)	
Age (in years)		
[5–10] (*n* = 528)	8 (1.5)	< 0.001
[10–15] (*n* = 571)	33 (5.8)	
Education level of houshold head or spouse		
No education (*n* = 276)	15 (5.4)	0.17
Primary (*n* = 673)	20 (3.0)	
Secondary (*n* = 150)	6 (4.0)	
Professional activities of household’s head		
No activity(*n* = 239)	8 (3.3)	0.832
Agriculture (*n* = 57)	1 (1.8)	
Seller (*n* = 536)	23 (4.3)	
Official (*n* = 190)	9 (3.3)	
Knowledge of the diseases		
Blood in urine and stool (*n* = 211)	11 (5.2)	0.224
Knowledge on transmission		
Drinking unsafe water (*n* = 44)	2 (4.6)	1.00
Washing in surface water (*n* = 111)	8 (7.2)	0.45
Contact with waste water (*n* = 87)	6 (6.9)	0.73

The environmental characteristics associated with *S. mansoni* cases are summarised in Table 3. A significant difference was found in the infection of children living in households where tap water (*P* = 0.045) and well water (*P* = 0.008) were the sources of drinking water. The difference is also pronounced considering the mode of drainage of solid waste (*P* = 0.026) and the proximity of the house to a surface water point (*P* = 0.001). Children living in households close to surface water points were 2.8 times more likely to be infected compared with those living far from water points.

**Table 3:** Environmental characteristics and their association with *S. mansoni* in Korhogo (March 2015)

Characteristics	Infected *n* (%)	*P*-value
Source of drinking water		
Tap water(*n* = 391)	21 (5.4)	0.045
Well water(*n* = 684)	17 (2.5)	0.008
Pump (*n* = 61)	3 (4.9)	0.493
Source of water for laundry		
Tap water (*n* = 214)	10 (4.7)	0.422
Well water (*n* = 880)	30 (3.4)	0.321
Surface water (*n* = 5)	0 (0.0)	1.000
Pump (*n* = 45)	2 (4.4)	0.680
Source of water for washing dishes		
Tap water (*n* = 238)	11 (4.6)	0.442
Well water (*n* = 845)	27 (3.2)	0.090
Surface water (*n* = 11)	0 (0.0)	1.000
Pump (*n* = 49)	2 (4.1)	0.703
Source of water for garden		
Tap water (*n* = 39)	2 (5.1)	0.650
Well water (*n* = 132)	8 (6.1)	0.140
Surface water (*n* = 11)	0 (0.0)	1.000
Pump (*n* = 7)	0 (0.0)	1.000
Type of latrine		
Modern latrine (*n* = 255)	9 (3.5)	0.850
Traditional latrine (*n* = 935)	34 (3.6)	0.331
No latrine (*n* = 52)	2 (3.8)	
Drainage of waste water		
Septic tank (*n* = 145)	7 (4.8)	0.164
In the nature (*n* = 954)	34 (3.6)	
Drainage of solid waste		
Garbage dump (*n* = 318)	13 (4.1)	0.026
In the nature (*n* = 780)	28 (3.6)	
House construction material		
Wood house (*n* = 148)	4 (2.7)	0.936
Brick home (*n* = 951)	37 (3.9)	
House light source		
Electricity (*n* = 838)	35 (4.2)	0.103
Artisanal source (*n* = 261)	6 (2.3)	
Existence of surface water		
Point at 500 m from house		
Yes (*n* = 392)	25 (6.4)	0.001
No (*n* = 703)	16 (2.3)	

### Univariate analysis and multiple regression analysis

Table 4 summarises the univariate and multiple regression analysis of risk factors of *S. mansoni* in Korhogo based on the surveyed sociodemographic and environmental factors. The risk of infection with *S. mansoni* increased with age (*OR* = 6.3; 95% *CI*: 2.30–17.20; per year). Girls were significantly less likely to be infected than boys (*OR* = 0.08; 95% *CI*: 0.03–0.26). Non-use of well water as source of drinking water in the household was strongly positively associated with the occurrence of *S. mansoni* (*OR* = 2.79; 95% *CI*: 1.20–6.51). Other factors, such as, abstaining from swimming in surface water (*OR* = 0.16; 95% *CI*: 0.04–0.56) and increased proximity of the household to water points (*OR* = 0.29; 95% *CI*: 0.13–0.70) were significantly negatively associated with *S. mansoni* infection. In the multiple logistic regression analysis, remoteness of a household from water points (*aOR* = 0.31; 95% *CI*: 0.12–0.82), not swimming in surface water (*aOR* = 0.12; 95% *CI*: 0.02–0.66) and education (*aOR* = 0.40; 95% *CI*: 0.16–0.99) showed a statistically significant protective association with *S. mansoni*. The respective ORs were very similar to those obtained without adjustment for age, sex and socioeconomic characteristics of the household. Thus, socioeconomic status was not a confounder of the observed associations.

**Table 4:** Univariable and multiple logistic regression analysis of variables associated with *S. mansoni* among study participants non-adjusted and adjusted for age and sex and for socioeconomic status of household head in Korhogo

Schistosomiasis	*OR* (95% *CI*)	a*OR* (95% *CI*)
Sociodemographic factors		
Age (in year) ([10–15]/[5–10])	6.26 (2.30–17.20)	6.05 (2.32–15.80)*
Sex (females / males)	0.08 (0.03–0.25)	0.08 (0.03–0.26)*
School level of the children^a^		
Schooled/non–schooled	0.63 (0.28–1.38)	0.40 (0.16–0.99)
School level of parents^b^		
High/low	1.14 (0.46–2.85)	1.37 (0.51–3.68)
Environmental factors (no vs. yes)		
Well as source of drinking water	2.79 (1.20–6.51)	2.93 (1.09–7.92)*
Well as source of plant watering	0.54 (0.18–1.60)	0.40 (0.11–1.48)
Tap as source of drinking water	0.47 (0.20–1.08)	0.45 (0.17–1.21)
Using modern latrine	1.16 (0.42–3.17)	1.27 (0.42–3.86)
Household at 500 m from surface water	0.29 (0.13–0.70)	0.31 (0.12–0.82)*
Swimming in surface water point	0.16 (0.04–0.56)	0.12 (0.02–0.66)*
Fishing in surface water	0.30 (0.07–1.31)	0.23 (0.01–7.93)
Socio–economic status (by PCA)^c^		
Most poor		
Poor	4.75 (1.20–18.88)	
Less poor	2.45 (0.70–8.58)	

*P < 0.05, *P-*value obtained from mixed logistic regression model with *S. mansoni* infection as outcome and household as clustering factor

^a^Schooled = Children educated in the classical system: primary and secondary school; Non-schooled = Children non-educated in the classical system: who never went to school + Koranic school

^b^High = Parents with university and secondary schools level; Low = parents with primary and Koranic school level or who never went to school

^c^Principal component analysis (PCA) based on the possession of: television, radio, fridge, bicycle, motorbike, car, electricity, ventilator, tap water, latrine, brick home

## Discussion

### Prevalence of schistosomiasis

In our survey of 728 households in March 2015, we found an overall prevalence of schistosomiasis among school-aged children in Korhogo of 2%, which is considered low for endemic communities according to WHO [27]. It is within the range of prevalence rates reported by Yapi et al. [28] in 1997–1999 in some villages of the same region (i.e. prevalence rates of 2.1–16.1% for *S. mansoni* and 0.7–4.8% for *S. haematobium)*. The authors, however, found the prevalence to be higher in western Côte d’Ivoire (a forest zone), in the order of 0.9–4.4% for *S. haematobium* and 17.5–61.3% for *S. mansoni* [28]. Data from southern Côte d’Ivoire suggest even higher prevalences in school-aged children (i.e. 58.7–68.4% and 10.9–18.4% for *S. mansoni* and *S. haematobium,* respectively) [29]. Although the risk of schistosomiasis still persists in northern Côte d’Ivoire, we have found that it was lower compared with other regions of the country for both forms of schistosomiasis. This may be due to the differences in environmental and ecology factors across the country [30]. Northern Côte d’Ivoire is the driest part of the country. It is a Savannah zone and the main economic activity is trade. In contrary, western and southern Côte d’Ivoire are wet zones. The main economic activity is agriculture [31]. Indeed the prevalence of schistosomiasis in Korhogo is more similar to the prevalence in Burkina Faso, bordering Côte d’Ivoire in the north and in close proximity to our study setting with similar environmental parameters. In 2013 in Burkina Faso, according to a national assessment, the prevalence of *S. mansoni* and *S. haematobium* were found to be 0–8.7% and 0–34.4%, respectively [31].

### Sociodemographic factors associated with *S. mansoni*

We found that boys were eight times more likely to be infected with *S. mansoni* than girls, which is different from the findings from southern Côte d’Ivoire [29] where no difference in infections was reported between males and females. However, it is likely that some socio-cultural and economic activities linked with the socioeconomic conditions and habits in Korhogo, such as watering cattle, fishing and swimming lead to higher exposure of males. In general, girls are at home or at market to help their mothers in the daily activities.

The prevalence of *S. mansoni* increased with age. Prevalence was higher in the older age group of 10–15 year-old, which is in line with previous studies conducted in south-western Ethiopia [32] and Nigeria [33]. This may be explained by the fact that children at this particular age group are more involved in household chores or recreational activities taking them further from home and potentially bringing them in contact with water.

The education level of the children was also strongly associated with the transmission of *S. mansoni,* with children in Koranic schools being more likely to be infected than others. The courses in Koranic schools are taken only in the morning, giving the children more free time for out-of-school activities. We likewise reported higher prevalence of *S. mansoni* in children in households where the head of the household was active in sales. Such parents are likely to be at the market all day, thus children may be less supervised and may spend more time around water points as part of their recreational activities.

### Environmental factors associated with *S. mansoni*

The results of our study indicated that associations between the type of latrine and *S. mansoni* infection were not significant in Korhogo. One of the explanations is that, in the transmission of *S. mansoni*, the infected intermediate host snails release cercariae in water, which may infect people that are exposed to the water. In this context, the type of latrine is not important as long as it fulfills its function of containing the human waste and thereby preventing the contamination of water bodies. Moreover, the study showed that the infection prevalence was high for children living in houses close to surface water access points and also for children who swim in these waters. The presence of a surface water points close to the house can facilitate human-water contact and increases exposure. People often have to cross open water to get to their destination, and it is commonly observed that persons defecate in or around these water points. Swimming also generally increases the risk of schistosomiasis in our study area. Children whose parents used a well as source of water for crops also showed higher prevalence rates compared with children whose parents did not. In Korhogo, urban agriculture is practiced in the shallows and around the water supply dam. To facilitate watering of crops, farmers often make wells around these water points. Furthermore, children of farmers are often engaged in watering. This potentially puts the children in constant direct contact with unhygienic water and increases the risk of infection.

Finally, the multiple regression analyses indicated that socioeconomic status of the head of household did not confound associations between behavioural and environmental risk factors and *S. mansoni* in Korhogo.

## Conclusion

The present study in northern Côte d’Ivoire found a low prevalence for urogenital and intestinal schistosomiasis among school-aged children. Sex, age, living near surface waters, swimming and irrigation of crops with well water were the significant determinant factors for infection by *S. mansoni*. However, the socioeconomic status was not related to the infection status. Despite our findings, efforts are needed to further reduce the prevalence of infection or to break transmission in this part of Côte d’Ivoire. Complementing praziquantel-based treatment with supplementary preventive measures such as health education for children and parents, especially those practicing urban agriculture and the provision of sanitary facilities is recommended. Taken together, reducing the risk of water contact seems an important component for an elimination strategy in this part of West Africa. An integrated approach combining infrastructural improvements and educational measures is urgently needed in this region. Moreover, it would be important to repeat the same study in the rainy season in Korhogo to assess the role of seasonality on the transmission of schistosomiasis.

## Additional file

Additional file 1: Multilingual abstracts in the six official working languages of the United Nations. (PDF 594 kb)

## Abbreviations

*CI*: Confidence interval
CSRS: Centre Suisse de Recherches Scientifiques en Côte d’Ivoire
*OR*: Odds Ratio
WASH: Water, Sanitation and Hygiene
WHO: World Health Organization
