# Supplementary material for: Risk factors for schistosomiasis in an urban area in northern Côte d’Ivoire
Source: Infect Dis Poverty. 2018 May 18;7:47. doi: 10.1186/s40249-018-0431-6 (PMC5958400; doi:10.1186/s40249-018-0431-6)
Supplement: Supplementary file 1 — Multilingual abstracts in the six official working languages of the United Nations. (PDF 594 kb) [file 40249_2018_431_MOESM1_ESM.pdf]

Translation of the abstract into the six official working languages of the United Nations

عوامل الخطر المتعلقة بداء البلهارسيا في منطقة داخل مدينة تقع في شمال ساحل العاج

قدمه: ريتشارد ك. مبرا، وبراما كوني، ويابي جي. يابي، وكغبافوغي دي سيلوي، إبراهيما سي، ودانييل فيينو، وناجين سورو، وجيلاديو سيسيه ويورغأ وتزينقر.

الخلاصة

المعلومات الأساسية: إن داء البلهارسيا هو أحد الأمراض المنقولة عن طريق المياه إذ ينتقل عبرالمثقوبات التي تنتمي إلى نوع البلهارسيا. تهدف هذه الدراسة إلى تقييم العلاقة التي تربط بين تفشي داء البهارسيا وإمكانية الحصول على المياه، والصرف الصحي والنظافة الشخصية (WASH) والعوامل الاجتماعية البيئية والاجتماعية الاقتصادية في مدينة كوروغو.

طرق البحث: أجريت دراسة شاملة تتضمن عينة من 728 أسرة اختيرت عشوائياً في مدينة كوروغو في شهر مارس 2015م. أجريت مقابلات مع أرباب الأسر التي اختيرت حول إمكانية الحصول على المياه، والصرف الصحي والنظافة الشخصية (WASH) والعوامل الاجتماعية البيئية والاجتماعية الاقتصادية المحيطة بهم. وقد وقع الاختيار على جميع الأطفال الذين تتراوح أعمارهم ما بين ٥ و١٥ عاماً ممن يعيشون مع الأسر المختارة لكي يوفروا عينات من البراز والبول بغرض تشخيصها بالإصابة بعدوى طفيليات البلهارسيا المنسونية والبلهارسيا الدموية. تم تحليل العلاقة التي تربط بين الإصابة بعدوى البلهارسيا المنسونيةوعوامل الخطر المحتملة باستخدام نموذج مختلط لتراجع النمو النسبي بوضع "الأسرة" عاملاً عشوائياً.

النتائج: كان مجمل نسبة انتشار داء البلهارسيات بين الأطفال في سن الدراسة بمدينة كوروغو .1.9٪ (2341/45) تتألف من 0.3٪ (1248/3) داء البلهارسيات البولي و 3.5٪ (1202/42) من داء البلهارسيات المعوي. بسبب انخفاض نسبة انتشار الإصابة بعدوى البلهارسيا الدموية، اقتصر تحليل عوامل الخطر إلىالبلهارسيا المنسونية. تعتبر فرص إصابة الصبيان بعدوى البلهارسيا المنسونيةأكثر بمعدل 7.8 مرات من الفتيات. الأطفال الذين تتراوح أعمارهم بين 10 و15 عاماً عرضة للإصابة بالعدوى بمعدل 3.8 مرات أكثر من الأطفال الذين تتراوح أعمارهم بين 5 و10 سنوات. وإضافةً إلى ذلك، أن العيش في منزل بعيد عن نقطة الوصول إلى مصدر المياه (نسبة الأرجحية [أو]=0.29، 95٪فاصل الثقة:0.13-0.70) والامتناع عن السباحة في المياه السطحية (أو=0.16، 95٪ فاصل الثقة:0.04-0.56) كلها عوامل ترتبط ارتباطاً وثيقاً بانخفاض احتمالات الإصابة بعدوى البلهارسيا المنسونية. إن الأطفال الذين لم يستخدم ذويهم مياه الآبار كمصدر لري النباتات يتمتعون بحماية ضد الإصابة بالبلهارسيا المنسونية (أو=0.54، 95٪ فاصل الثقة: 0.18-1.60). إلا أن الوضع الاجتماعي الاقتصادي لم يكن مؤثرًا على ما يبدو في تفشي البلهارسيا المنسونية.

الخاتمة: ينبغي أن تركز الاستراتيجية المستدامة لخفض حالات الإصابة بالبلهارسيا على التوعية الصحية لتغيير سلوك السكان المعرضين للخطر وتشجيع المجتمعات على تحسين الصرف الصحي والبنية التحتية بهدف الحد من ملامسة المياه السطحية.

Translated from English version into Arabic by Bashaier Allam, proofread by Suzan Alkhodair, through

TRANSLATORS
WITHOUT BORDERS

科特迪瓦北部城市地区血吸虫病的风险因素

Richard K. M’Bra, Brama Kone, Yapi G. Yapi, Kigbafori D. Silué, Ibrahima Sy, Danielle Vienneau, Nagnin Soro, Guéladio Cissé and Jürg Utzinger

摘要

引言：血吸虫病是一种由血吸虫属吸虫传播的水源性疾病。本研究旨在评估科霍戈市血吸虫病流行情况与水、环境卫生和个人卫生（water, sanitation and hygiene，WASH）可及性，以及社会环境和社会经济因素的相关性。

方法：2015年3月，研究人员在科霍戈市对随机抽取的728个家庭进行了横断面研究。询问各户主关于WASH可及性、社会环境和社会经济因素等方面的情况。采集所有家庭中5–15岁儿童的粪样和尿样，进行曼氏血吸虫和埃及血吸虫感染的病原学诊断。以“家庭”作为随机变量，应用混合逻辑回归模型分析曼氏血吸虫感染与潜在风险因素之间的关系。利用似然比检验确定与血吸虫感染显著相关的因素。
结果：科霍戈市学龄儿童血吸虫病的总体患病率为1.9％（45/2341），尿路血吸虫病和肠血吸虫病的患病率分别为0.3％（3/1248）和3.5％（42/1202）。考虑到埃及血吸虫感染率较低，危险因素分析仅限于曼氏血吸虫。结果显示，男生感染曼氏血吸虫的概率是女生的7.8倍。10–15岁儿童感染曼氏血吸虫的概率是5–10岁儿童的3.8倍。居住地远离取水点[比值比(*OR*)=0.29, 95% *CI*: 0.13–0.70)、不在地表水中游泳(*OR*=0.16, 95% *CI*: 0.04–0.56)与曼氏血吸虫感染概率下降显著相关。父母不使用井水灌溉植物的儿童感染曼氏血吸虫的概率也较低（*OR*=0.54, 95％ *CI*：0.18–1.60）。然而，社会经济地位似乎对曼氏血吸虫的流行影响不大。
结论：降低血吸虫病发病率的可持续战略应侧重于健康教育，改变高危人群的行为，同时鼓励社区改善环境卫生和基础设施，以减少与地表水的接触。

Translated from English version into Chinese by Fan Yang, edited by Pin Yang

Factores de riesgo de esquistosomiasis en un área urbana en el norte de Côte d'Ivoire

Richard K. M’Bra, Brama Kone, Yapi G. Yapi, Kigbafori D. Silué, Ibrahima Sy, Danielle Vienneau, Nagnin Soro, Guéladio Cissé and Jürg Utzinger

Resumen

Introducción: La esquistosomiasis es una enfermedad de transmisión hídrica por trematodos pertenecientes al género*Schistosoma* El objetivo de este estudio fue evaluar la relación entre la prevalencia de esquistosomiasis y el acceso al suministro de agua, al saneamiento e higiene (WASH) y a factores socio-ambientales y socioeconómicos en la ciudad de Korhogo.

Métodos: En marzo se 2015 se llevó a cabo en Khorgo un estudio transversal que incluyó a 728 hogares seleccionados al azar. Se entrevistó a los jefes de hogar sobre el acceso a WASH y de los factores socio-ambientales y socioeconómicos. Se seleccionó a todos los niños y jóvenes entre 5 y 15 años de edad que vivían en esos hogares para que proporcionaran muestras de materia fecal y orina para estudiar la posible presencia de infección por *S. mansoni* y *S. haematobium*. La relación entre la infección con *S. mansoni* y los posibles factores de riesgo se analizó mediante un modelo de regresión logística mixto con el factor «hogar» como factor aleatorio. Se utilizaron pruebas de cociente de probabilidad para identificar los factores asociados significativamente con una infección por *Schistosoma* spp.

Resultados: La prevalencia general de la esquistosomiasis entre los niños en edad escolar, en Korhogo fue 1,9 % (45/2341), compuesto por un 0,3 % (3/1248) al esquistosomiasis urinaria y 3,5 % (42/1202) a la esquistosomiasis intestinal. Debido a la baja prevalencia de la infección por *S. haematobium*, los análisis de los factores de riesgo se limitaron a *S. mansoni*. Los jóvenes mostraron una propensión 7,8 veces mayor a infectarse con *S. mansoni* que las jóvenes. Los jóvenes entre 10 y 15 años mostraron una propensión 3,8 veces mayor a infectarse que los niños de 5 a 10 años. Por otro lado, el hecho de residir en una vivienda alejada de un punto de acceso al agua (tasa de probabilidad (Odds Ratio)[*OR*] =0,29, 95 % *IC*: 0,13-0,70) y de abstenerse de nadar en aguas superficiales (*OR*=0,16, 95 % *IC*: 0,04-0,56) se asociaron significativamente con la disminución de la probabilidad de infección por *S. mansoni*. Los niños y jóvenes

cuyos padres no usaron el agua de pozo como fuente para el riego de las plantas estuvieron mejor protegidos contra *S. mansoni* (*OR*=0,54, 95 % *IC*: 0,18-1,60). Sin embargo, el factor socioeconómico no pareció influir en la prevalencia de *S. mansoni*.

Conclusion: Una estrategia sostenible para reducir la incidencia de la esquistosomiasis debería centrarse en la educación sanitaria para cambiar el comportamiento de las poblaciones en riesgo, y alentar a las comunidades a mejorar el saneamiento y la infraestructura a fin de reducir el contacto con las aguas superficiales.

Translated from English version into French by Juan José Moreiras, proofread by Ana Vanoli, through

Факторы риска распространения шистосомоза в одном из городских районовсеверного Кот-д'Ивуара

Ричард К. М'Бра (Richard K. M'Bra), Брама Кон (Brama Kone), Япи Г. Япи (Yapi G. Yapi) , Кигбафори Д. Силью (Kigbafori D. Silué) , Ибрахима Сай (Ibrahima Sy), Даниэль Вьенно (Danielle Vienneau), Нагнин Соро (Nagnin Soro), Геладио Циссе (Guéladio Cissé) и Юрг Утзингер (Jürg Utzinger)

Аннотация

Справочная информация: Шистосомоз - это заболевание, передающееся через воду трематодами, которые относятся к роду *Schistosoma* . Цель данного исследования - оценить взаимосвязь между распространенностью шистосомоза и водоснабжением, санитарией и гигиеной, а также социально-экологическими и социально-экономическими факторами в городе Корхого.

Методы: В городе Корхого в марте 2015 года было проведено кросс-секционное исследование 728 домашних хозяйств, выбранных случайным образом. Главам этих семей были заданы вопросы по поводу водоснабжения, санитарии и гигиены, а также социально-экологических и социально-экономических факторов. У всех детей в возрасте от 5 до 15 лет, проживающих в данных семьях были взяты пробы кала и мочи для паразитологического исследования на инфекции *S. mansoni* и *S. haematobium*. Взаимосвязь между инфекцией *S. mansoni* и потенциальными факторами риска была проанализирована с помощью модели смешанной логистической регрессии, случайным фактором которой является "домашнее хозяйство". Для определения факторов, которые были существенно ассоциированы с инфекций *Schistosoma* spp., использовались тесты отношения правдоподобия.

Результаты: Общая распространенность шистосомоза среди детей школьного возраста в Корхого - 1,9% (45/2341), из которых 0,3% (3/1248) - мочеполовой шистосомоз, а 3,5% (42/1202) - кишечный шистосомоз. Из-за низкой распространенности инфекции *S. haematobium*, анализ факторов риска был произведен только для инфекции *S. mansoni*. Вероятность заражения инфекцией *S. mansoni* в 7,8 раза выше для мальчиков, чем для девочек. Вероятность инфицирования в 3,8 раза выше для детей в возрасте 10-15 лет, чем для детей в возрасте от 5 до 10 лет. К тому же, проживание в доме, который находится дальше от источника воды (отношение шансов [*ИЛИ*] =0.29, 95% *CI*: 0.13–0.70) , а также воздержание от купания в поверхностных водах (*ИЛИ*=0.16, 95% *CI*: 0.04–0.56), были существенно ассоциированы со снижением риска заражения инфекцией *S. mansoni*. Дети, родители которых не использовали колодезную воду для орошения растений, были более защищены от

инфекции *S. mansoni* (*ИЛИ* =0.54, 95% *CI*: 0.18–1.60). Однако, похоже, что социально-экономический статус не повлиял на распространенность инфекции *S. mansoni*.
Выводы: Долгосрочная стратегия сокращения случаев шистосомоза должна быть основана на санитарном просвещении, которое приведет к изменениям в поведении населения в группе риска, и пробудит желание улучшить санитарию и инфраструктуру для уменьшения контакта с поверхностными водами.

Translated from English version into Russian by Oksana Rozhko, proofread by Ekaterina_Rugg, through

Factores de riesgo de esquistosomiasis en un área urbana en el norte de Côte d'Ivoire

Richard K. M'Bra, Brama Kone, Yapi G. Yapi, Kigbafori D. Silué, Ibrahima Sy, Danielle Vienneau, Nagnin Soro, Guéladio Cissé and Jürg Utzinger

Resumen

Introducción: La esquistosomiasis es una enfermedad de transmisión hídrica por trematodos pertenecientes al género*Schistosoma* El objetivo de este estudio fue evaluar la relación entre la prevalencia de esquistosomiasis y el acceso al suministro de agua, al saneamiento e higiene (WASH) y a factores socio-ambientales y socioeconómicos en la ciudad de Korhogo.
Métodos: En marzo se 2015 se llevó a cabo en Khorgo un estudio transversal que incluyó a 728 hogares seleccionados al azar. Se entrevistó a los jefes de hogar sobre el acceso a WASH y de los factores socio-ambientales y socioeconómicos. Se seleccionó a todos los niños y jóvenes entre 5 y 15 años de edad que vivían en esos hogares para que proporcionaran muestras de materia fecal y orina para estudiar la posible presencia de infección por *S. mansoni* y *S. haematobium*. La relación entre la infección con *S. mansoni* y los posibles factores de riesgo se analizó mediante un modelo de regresión logística mixto con el factor «hogar» como factor aleatorio. Se utilizaron pruebas de cociente de probabilidad para identificar los factores asociados significativamente con una infección por *Schistosoma* spp.
Resultados: La prevalencia general de la esquistosomiasis entre los niños en edad escolar, en Korhogo fue 1,9 % (45/2341), compuesto por un 0,3 % (3/1248) al esquistosomiasis urinaria y 3,5 % (42/1202) a la esquistosomiasis intestinal. Debido a la baja prevalencia de la infección por *S. haematobium*, los análisis de los factores de riesgo se limitaron a *S. mansoni*. Los jóvenes mostraron una propensión 7,8 veces mayor a infectarse con *S. mansoni* que las jóvenes. Los jóvenes entre 10 y 15 años mostraron una propensión 3,8 veces mayor a infectarse que los niños de 5 a 10 años. Por otro lado, el hecho de residir en una vivienda alejada de un punto de acceso al agua (tasa de probabilidad (Odds Ratio)[*OR*] =0,29, 95 % *IC*: 0,13-0,70) y de abstenerse de nadar en aguas superficiales (*OR*=0,16, 95 % *IC*: 0,04-0,56) se asociaron significativamente con la disminución de la probabilidad de infección por *S. mansoni*. Los niños y jóvenes cuyos padres no usaron el agua de pozo como fuente para el riego de las plantas estuvieron mejor protegidos contra *S. mansoni* (*OR*=0,54, 95 % *IC*: 0,18-1,60). Sin embargo, el factor socioeconómico no pareció influir en la prevalencia de *S. mansoni*.

Conclusion: Una estrategia sostenible para reducir la incidencia de la esquistosomiasis debería centrarse en la educación sanitaria para cambiar el comportamiento de las poblaciones en riesgo, y alentar a las comunidades a mejorar el saneamiento y la infraestructura a fin de reducir el contacto con las aguas superficiales.

Translated from English version into Spanish by Juan José Moreiras, proofread by Ana Vanoli, through

TRANSLATORS
WITHOUT BORDERS
